# EchoGO: annotation-context-aware functional interpretation of transcriptomic data

**DOI:** 10.1016/j.mex.2026.104222

**Published:** 2026-10-22

**Authors:** Camilo Escobar-Sierra, Felix Langschied, Angelina Miller, Pedro A. Inostroza

**Affiliations:** a Chair of Ecotoxicology and Environmental Risk Assessment, Institute for Environmental Research, RWTH Aachen University, Aachen, Germany; b Institute of Cell Biology and Neuroscience, Applied Bioinformatics Group, Goethe University Frankfurt, Frankfurt am Main, Germany; c Department of Biological and Environmental Science, University of Gothenburg, Gothenburg, Sweden

**Keywords:** Functional enrichment, Transcriptomics, Non-model organisms, Annotation context, Functional interpretation, Gene Ontology, Reproducible workflow

## Abstract

Functional enrichment of transcriptomic datasets can be constrained by incomplete annotation, uneven identifier recognition, and uncertainty about which organism annotation resource provides the most informative functional context. EchoGO is a reproducible downstream framework that retains target GOseq enrichment as the primary functional evidence while evaluating the same transcriptomic response across researcher-selected organism annotation contexts using experiment-specific backgrounds. Evidence is assembled at the GO-term level with its analytical and organism provenance, allowing researchers to distinguish functions supported by the target analysis, functions also recovered through alternative annotation contexts, and additional context-derived hypotheses. Semantic reduction, gene-sharing networks, visualisation, and reporting organise this evidence into interpretable biological summaries. Validation across a reference-rich zebrafish dataset and two annotation-limited *Gammarus pulex* comparisons revealed contrasting patterns of annotation-context dependence. In zebrafish, 42 of 44 target GO terms were also recovered through alternative fish contexts, while additional contexts expanded the range of functional hypotheses. In *G. pulex*, limited identifier representation restricted contextual analysis; recovered functions were predominantly translation-related in one comparison and also included contractile biology in the other. EchoGO provides a traceable framework for examining how annotation availability and organism context shape functional interpretation while preserving the target analysis as the experimental anchor.

## Graphical abstract

**Figure:**
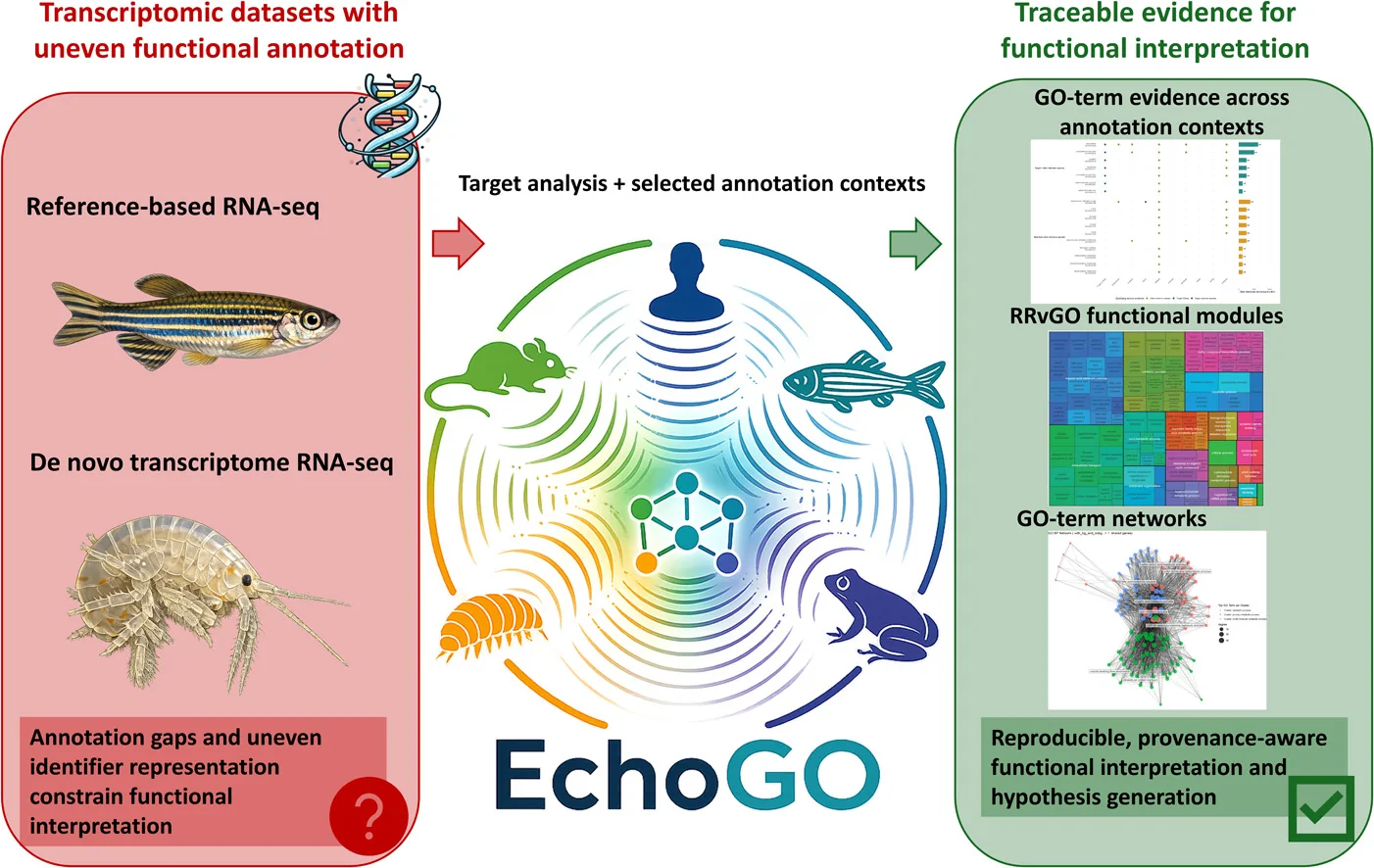

Specifications table

**Table:** 

**Subject area**	Bioinformatics
**More specific subject area**	Functional enrichment and annotation-context-aware interpretation of transcriptomic data
**Name of your method**	EchoGO
**Name and reference of original method**	Not applicable. EchoGO is a functional-interpretation workflow that integrates established methods for RNA-seq enrichment, contextual functional analysis, and semantic reduction, including GOseq, g:Profiler/gprofiler2, and RRvGO.
**Resource availability**	EchoGO source code and documentation: https://github.com/miloes114/EchoGo | EchoGO v0.1.4: https://doi.org/10.5281/zenodo.23158481 | Validation and reproducibility compendium v2.0.0: https://doi.org/10.5281/zenodo.23164928

## Background

Functional enrichment is central to the biological interpretation of transcriptomic datasets, yet interpretation remains challenging when genomic resources are incomplete, annotation coverage is uneven or taxonomically biased, or available annotation resources are poorly matched to the biological question. These limitations are particularly evident in non-model organisms, but annotation context can also influence interpretation in reference-based studies. The challenge is therefore not only to identify differentially expressed genes, but to translate these responses into biologically meaningful functions while making the influence of annotation resources on their interpretation visible [1,2].

Functional enrichment of RNA-seq data also requires methodological care because the probability of detecting differential expression is not random with respect to transcript properties such as length. GOseq was developed to account for this source of bias and remains an important component of enrichment analysis for RNA-seq data [3]. Complementary resources extend this analytical landscape: eggNOG-mapper supports functional annotation and orthology-based interpretation, while g:Profiler and its R interface gprofiler2 provide enrichment analysis across many organisms [4,5]. Because enrichment analyses can produce long and redundant Gene Ontology term lists, semantic-reduction approaches such as RRvGO can condense these outputs into more interpretable functional themes [6].

Together, these resources provide a strong foundation for transcriptomic interpretation, but functional knowledge remains unevenly distributed among organisms and annotation databases. Experimental characterisation of gene function is concentrated in model organisms [7], and functional annotations are commonly transferred to non-model organisms based on the orthology–function conjecture [8]. Such transfer is informative, but orthologs do not necessarily retain identical functions across evolutionary lineages, and functional divergence can become increasingly relevant across large evolutionary distances [9]. Reference-species selection can also propagate into downstream functional interpretation. In non-model transcriptomes, Pai et al. [10] found substantial differences in functional annotation among candidate reference species and recovered additional significant GO terms and pathways when multiple reference species were used for annotation and enrichment. Their results also illustrate that phylogenetic or sequence-resource suitability and functional annotation depth need not coincide. This challenge is illustrated by *Gammarus pulex*, a widely used non-model sentinel in freshwater ecotoxicology whose functional interpretation often relies on information from better-characterised model organisms [[11], [12], [13], [14]]. For example, amphipod and insect lineages, including *G. pulex* and *Drosophila melanogaster*, diverged more than 500 million years ago [15]. More generally, the functional picture recovered from a transcriptomic response can therefore depend partly on the annotation context through which that response is examined.

EchoGO was developed to make annotation-context dependence explicit during functional interpretation. It retains the study-specific functional analysis of the experimental dataset (hereafter, the target analysis) as the primary evidence and examines the same transcriptomic response through researcher-selected organism annotation resources. Here, the target organism refers to the species studied experimentally, whereas an annotation context refers to an organism-specific functional annotation resource used to evaluate gene identifiers resolved from the experimental dataset. These contexts may correspond to the target organism itself or to other organisms selected for their biological or analytical relevance. Functional evidence is assembled at the GO-term level while retaining its analytical and organism provenance, allowing researchers to distinguish functions supported only by the target analysis, target-supported functions also recovered through alternative annotation contexts, and additional context-derived hypotheses for further investigation. By linking these patterns to identifier representation, organism-specific recognition, and the annotation resources from which they arise, EchoGO provides a traceable framework for examining how annotation context shapes the biological interpretation of transcriptomic responses.

## Method details

### Overview and intended use

Building on this rationale, EchoGO begins after differential-expression analysis and can be applied to both de novo and reference-based RNA-seq studies. Fig. 1 summarises this logic, following the experimental feature space through two complementary analytical routes: the study-specific target analysis and contextual enrichment across researcher-selected organism annotation resources, which subsequently converge at the level of GO-term evidence.

**Fig. 1:**
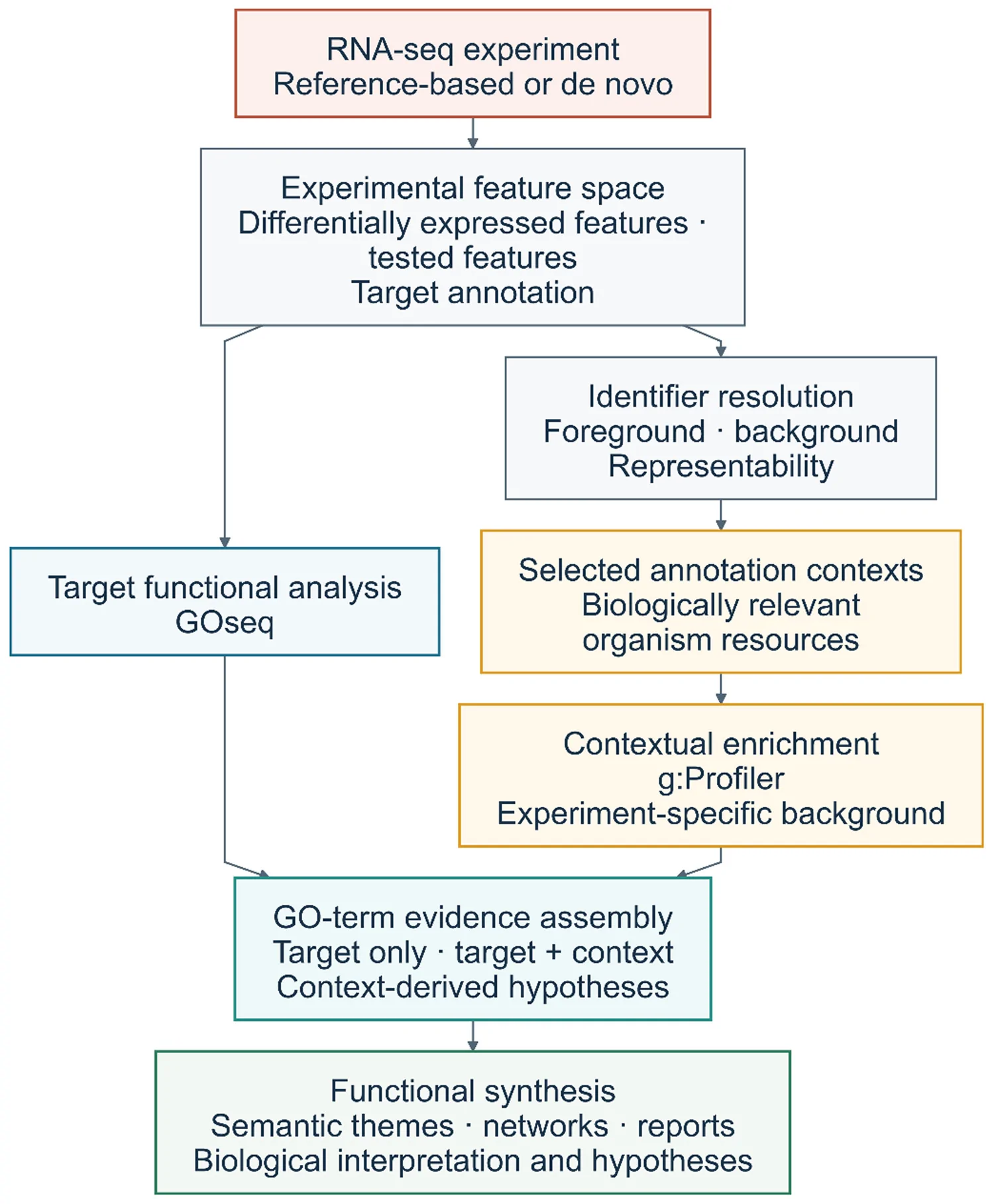
**EchoGO workflow for annotation-context-aware functional interpretation.** EchoGO retains the target GOseq analysis while resolving the experimental foreground and tested-feature background for contextual enrichment across researcher-selected organism annotation resources. The target and contextual routes converge at individual Gene Ontology (GO) terms, preserving analytical source, annotation context, and source-specific statistical evidence. Evidence assembly distinguishes functions supported only by the target analysis, target-supported functions also recovered through alternative contexts, and context-derived hypotheses. Semantic grouping, gene-sharing networks, and reports support biological interpretation while retaining this provenance. Optional default-domain analyses form a separate exploratory layer and are omitted from the primary workflow shown here. Features refer to the documented analysis units of each study.

For each comparison, EchoGO operates on the experimental features used for differential-expression analysis. Here, a feature refers to the unit quantified and tested for differential expression, such as an annotated gene in a reference-based study or a gene-level unit from a de novo transcriptome assembly. The workflow requires differential-expression results, the complete set of tested features, corresponding annotation mappings, and precomputed GOseq enrichment. Features meeting the study's differential-expression criterion define the foreground, whereas the complete set of features included in differential-expression testing, including those not classified as differentially expressed, defines the experiment-specific background. For contextual enrichment, foreground and background features are processed through the same identifier-resolution procedure to generate matched portable gene sets. This resolution step belongs to the contextual branch of the workflow; features contributing to the target GOseq analysis remain traceable even when no portable identifier suitable for contextual analysis can be resolved.

In the contextual branch of Fig. 1, the resolved foreground and background are evaluated with g:Profiler across researcher-selected organism annotation contexts using the experiment-specific background. The same resolved gene sets are submitted to every selected context, while organism-specific recognition determines the effective query and annotation domain within each resource. In parallel, the target branch retains the precomputed GOseq results based on the study-specific gene-to-GO annotation and RNA-seq bias correction. The two branches therefore address complementary aspects of the same transcriptomic response and are not combined statistically; instead, they converge at the GO-term evidence-assembly stage shown in Fig. 1.

At this convergence point, EchoGO assembles individual GO terms while retaining their analytical source, organism annotation context, source-specific statistics, and background mode. Primary evidence is organised into three categories—Target only, Target + context, and Context-derived hypotheses—according to whether qualifying GO-term evidence comes from target GOseq, alternative annotation contexts, or both. Optional g:Profiler analyses using its default organism-wide domain remain a separate exploratory layer. As illustrated in the final part of Fig. 1, the assembled term-level evidence can then be organised through semantic reduction, gene-sharing networks, visualisations, and reports to support biological interpretation without losing the provenance of the underlying evidence.

### Software availability

EchoGO is implemented as an R workflow with versioned source code, documentation, demonstration data, and reproducible analysis outputs. Input files can be provided from user-defined locations without requiring a fixed project directory structure. Versioned releases and archived validation materials are provided through GitHub and Zenodo.

### Input requirements and preparation

EchoGO requires four types of study-derived input for each transcriptomic comparison: differential-expression results identifying the foreground features, the complete set of features tested in that analysis, annotation mappings connecting experimental feature identifiers to functional identifiers, and the corresponding target GOseq enrichment results. Table 1 specifies the information required from each input. These requirements are independent of the upstream RNA-seq pipeline and its file-naming conventions.

**Table 1:** Analytical inputs required by EchoGO and their role in functional interpretation.

Input	Required information	Purpose
Differential-expression results	Experimental feature identifiers and differential-expression statistics for each comparison	Defines the foreground according to the user-defined differential-expression criterion
Tested features	All features included in differential-expression testing, including non-significant features	Defines the experiment-specific background for contextual enrichment
Annotation information	Mappings linking experimental identifiers to functional annotations and identifiers suitable for downstream analysis	Supports target interpretation, identifier resolution, and traceability
Target GOseq results	Precomputed GO-term enrichment results for the target dataset	Provides the primary target functional evidence

The tested-feature set defines the experiment-specific background used throughout contextual enrichment. Foreground and background identifiers are resolved using the same mapping procedure, drawing on available study annotations such as Swiss-Prot-derived gene symbols, eggNOG preferred names, or suitable native gene symbols. Resolution is performed consistently across the two sets, duplicates are collapsed before submission, and unresolved features remain documented in the analysis provenance. EchoGO records the submitted foreground and background vectors so that representability can be quantified relative to the original experimental feature sets.

The resolved vectors provide a common input for all selected organism annotation contexts. Because organism databases differ in identifier recognition and annotation coverage, the number of submitted genes recognised in a particular context can vary even when the input vectors are identical. EchoGO records these context-specific effective query and annotation-domain sizes alongside the enrichment results, allowing downstream interpretation to distinguish the experimental gene-set definition from representation within each annotation resource.

### Selection of organism annotation contexts

Organism annotation contexts are selected according to the biological and analytical purpose of the study. Relevant considerations can include taxonomic relationship to the target organism, ecological or physiological relevance, established model-organism use, and the availability and depth of functional annotation. These criteria need not identify the same species: previous work has shown that reference species with extensive sequence resources can differ substantially in functional annotation coverage [10]. EchoGO therefore records the selected contexts as part of the analysis configuration and provenance so that their biological rationale remains explicit.

Each selected context provides a distinct view of how its organism-specific annotation resource represents the same resolved experimental foreground and background. When a g:Profiler analysis uses the annotation resource of the organism studied experimentally, it is designated the target annotation context; resources corresponding to other organisms are designated alternative annotation contexts. Importantly, the target annotation context is distinct from the study-specific GOseq target analysis. EchoGO records the number of alternative annotation contexts recovering the same exact GO term as recurrence. This is a descriptive measure of annotation-context representation, not independent experimental confirmation or combined statistical significance.

### Target and contextual functional enrichment

EchoGO combines two complementary routes of functional evidence. The target route consists of the precomputed GOseq analysis, which evaluates enrichment using the study-specific gene-to-GO annotation while accounting for RNA-seq selection bias [3]. These results provide the primary functional evidence associated directly with the experimental dataset and remain distinct from contextual g:Profiler analyses, including any analysis performed using the target organism's own annotation resource.

The contextual route evaluates the resolved foreground against each selected organism annotation context using g:Profiler through gprofiler2 [5]. The experiment-specific resolved background is supplied as a custom background for each analysis. The submitted foreground and background vectors are held constant across organism contexts, while identifier recognition and functional annotation are determined by each organism-specific resource. Identifiers not recognised within a given organism context are excluded from the effective statistical domain used for that enrichment analysis. EchoGO records the submitted vectors, their identifiers and hashes, analysis parameters, and the effective query and annotation-domain sizes returned for each context.

Statistical significance is evaluated within each source analysis using its corresponding multiple-testing procedure. GOseq and each organism-specific g:Profiler analysis therefore retain their own adjusted *p*-values, enrichment measures, and statistical assumptions. EchoGO carries these source-level statistics into the term-level evidence record, where they remain associated with the analysis that produced them.

An optional exploratory route runs g:Profiler using its default organism-wide annotation domain. These analyses can expose additional functions for hypothesis generation when experiment-specific background representation is limited. They are retained as a separate exploratory evidence layer, with their background mode explicitly recorded in the provenance.

### Assembly and interpretation of functional evidence

EchoGO assembles qualifying GO-term results from the target and contextual analyses into a common term-level evidence structure. Each occurrence retains the GO identifier and ontology, analytical source, organism annotation context, background mode, source-specific statistical evidence, and associated gene information. This provenance allows individual functional findings to be traced from the integrated output to the enrichment analysis and annotation resource from which they originated.

Primary interpretation is organised into three evidence categories according to where qualifying GO-term evidence is recovered. Target only identifies GO terms supported by the study-specific GOseq analysis but not recovered through the selected alternative annotation contexts. Target + context identifies GO terms supported by GOseq and also recovered through at least one alternative annotation context. The Context-derived hypotheses category comprises GO terms recovered through one or more alternative annotation contexts without qualifying evidence from the target GOseq analysis. Here, recovery means that a GO term meets the significance criterion within a particular enrichment analysis; non-recovery does not establish biological absence. These categories describe the provenance of functional evidence, not a hierarchy of statistical confidence.

For terms recovered through contextual analysis, EchoGO records recurrence as the number of selected alternative organism annotation contexts in which the same exact GO term is recovered. This measure helps distinguish findings restricted to a single annotation resource from those recovered through several resources, without treating these resources as independent experimental replicates. The underlying source-local statistics and organism identities remain available alongside this summary, allowing recurrence to be interpreted in relation to the composition and annotation properties of the selected contexts.

Results from optional default-domain g:Profiler analyses are retained in a separate exploratory layer. Their provenance and source-level statistics remain available for hypothesis generation and inspection alongside the primary background-aware evidence.

### Functional synthesis and outputs

EchoGO provides complementary outputs for examining functional evidence at different levels of biological organisation. Term-level tables retain the complete evidence and provenance record, allowing individual GO terms to be inspected together with their supporting analyses, organism contexts, associated genes, and source-specific statistics. Ontology-level visualisations summarise the distribution of evidence across Biological Process, Molecular Function, and Cellular Component.

Because GO enrichment commonly produces groups of semantically related terms, EchoGO uses RRvGO to organise eligible GO terms into broader functional clusters based on semantic similarity [6]. Semantic reduction provides a higher-level view of related functional signals while retaining links to the underlying GO terms and their evidence categories. Representative terms can then be used to describe these clusters in figures and reports.

EchoGO additionally constructs gene-sharing networks in which GO terms are connected according to overlap in their associated gene sets. These networks provide a complementary view of relationships among enriched functions that arise from shared experimental genes rather than semantic similarity alone. Together, semantic grouping and gene-sharing networks help organise complex enrichment outputs into interpretable functional structures while preserving access to the underlying term-level evidence.

The workflow produces standardised tables, visualisations, semantic-reduction products, networks, diagnostic summaries, and optional HTML reports. Outputs retain the information required to trace biological interpretations to the experimental features, enrichment source, organism annotation context, and background definition from which they arose.

## Method validation

### Validation across contrasting annotation settings

EchoGO was evaluated using three RNA-seq comparisons representing different combinations of target-organism annotation depth and contextual identifier recognition. The first exercise (ZEB) used a reference-based zebrafish dataset and provided a reference-rich case in which the target organism is extensively represented in functional databases. Two additional exercises (GAM1 and GAM2) used *G. pulex* field transcriptomic comparisons derived from a de novo transcriptome and provided annotation-limited cases with substantially lower representation of the experimental feature space. Together, these exercises were used to examine two complementary aspects of annotation-context dependence: how much of the experimental feature space could be represented and recognised in the selected organism resources, and how the resulting functional evidence was distributed between the study-specific GOseq analysis and alternative annotation contexts.

Each exercise retained its original differential-expression results, tested-feature universe, annotation information, and target GOseq analysis. Foreground and background features were processed through the same identifier-resolution procedure described above, and the resulting matched vectors were submitted across the selected organism annotation contexts. For ZEB, the contextual analysis comprised zebrafish and eight additional fish annotation contexts. GAM1 and GAM2 were evaluated using six alternative invertebrate annotation contexts. The composition of each set reflected the biological and analytical context of the corresponding exercise and was recorded as part of the analysis provenance.

Evaluation considered both the representation of the experimental gene sets and the resulting GO-term evidence. Representability was evaluated from the number of unique portable gene identifiers obtained after resolution and deduplication of the tested-feature background and differentially expressed foreground, expressed relative to the corresponding original feature-set sizes. Context-specific recognition was additionally recorded from the effective query and annotation-domain sizes returned for each organism resource. Functional evidence was summarised using the three primary categories defined above: Target only, Target + context, and Context-derived hypotheses. The validation settings, gene-set representability, and resulting GO-term evidence for the three exercises are summarised in Table 2. Biological interpretation considered the GO terms recovered across annotation contexts, their associated genes, and the broader functional themes identified through semantic reduction.

**Table 2:** Validation settings, gene-set representability, and GO-term evidence across annotation contexts.

Validation feature	Zebrafish (ZEB)	*G. pulex* GAM1	*G. pulex* GAM2
Biological comparison	Chemically exposed embryos vs matched controls	Downstream field site vs upstream comparator	First downstream field site vs upstream comparator
Annotation setting	Reference-rich	Annotation-limited	Annotation-limited
Tested target features	21,004	150,407	150,407
Significant target features	115	2035	2093
Resolved background submitted	14,605 (69.53%)	5171 (3.44%)	5171 (3.44%)
Resolved foreground submitted	92 (80.00%)	239 (11.74%)	188 (8.98%)
Target GOseq terms	44	194	59
Target only	2	178	34
Target + context	42	16	25
Context-derived hypotheses	180	19	20
Selected annotation contexts	1 target + 8 alternative fish contexts	6 alternative invertebrate contexts	6 alternative invertebrate contexts
Default-domain exploration	Separate exploratory layer	Separate exploratory layer	Separate exploratory layer

Tested features refer to the entities included in the corresponding differential-expression analysis; for the *G. pulex* exercises, these are Trinity gene-level (_g) features rather than a biological estimate of the number of genes in the organism. Resolved foreground and background values represent unique portable identifiers submitted to contextual g:Profiler analysis after identifier resolution and deduplication. Percentages express these submitted identifier counts relative to the corresponding original significant or tested feature totals, respectively. GO-term counts refer to unique exact GO identifiers. Target only comprises GO terms supported by target GOseq without qualifying recovery through alternative annotation contexts; Target + context comprises target GOseq terms also recovered through at least one alternative annotation context; and Context-derived hypotheses comprise GO terms recovered through alternative annotation contexts without qualifying target GOseq evidence. All three categories use background-aware enrichment evidence. Recurrence describes the number of selected alternative annotation contexts in which an exact GO term was recovered. Default-domain exploratory results are reported separately from these primary evidence categories.

### Reference-rich zebrafish exercise

The zebrafish exercise provided the reference-rich validation setting. Identifier resolution yielded 14,605 unique background identifiers from 21,004 tested features (69.53% relative to the tested-feature total) and 92 unique foreground identifiers from 115 differentially expressed features (80.00%; Table 2). The study-specific GOseq analysis identified 44 significant GO terms, of which 42 were also recovered through at least one alternative fish annotation context (Target + context), leaving only two in the Target only category. The alternative annotation contexts additionally recovered 180 GO terms classified as Context-derived hypotheses.

The target GOseq response included functions associated with oxygen sensing and hypoxia, dioxygenase and oxidoreductase activity, and muscle and contractile biology. Oxygen-related evidence included genes such as *EGLN1–3, NDRG1*, and *LDHA*, whereas the muscle-associated response included genes such as *ACTN2, STAC3, SIX1, TTN*, and *MYOT*. Fig. 2A illustrates how representative terms from these target functions were distributed across the selected annotation contexts. Muscle cell differentiation was recovered through five alternative fish contexts and response to decreased oxygen levels through four, illustrating that prominent components of the target response were represented across several selected fish annotation resources. In contrast, response to insulin, represented in Fig. 2A, belonged to the Target only category.

**Fig. 2:**
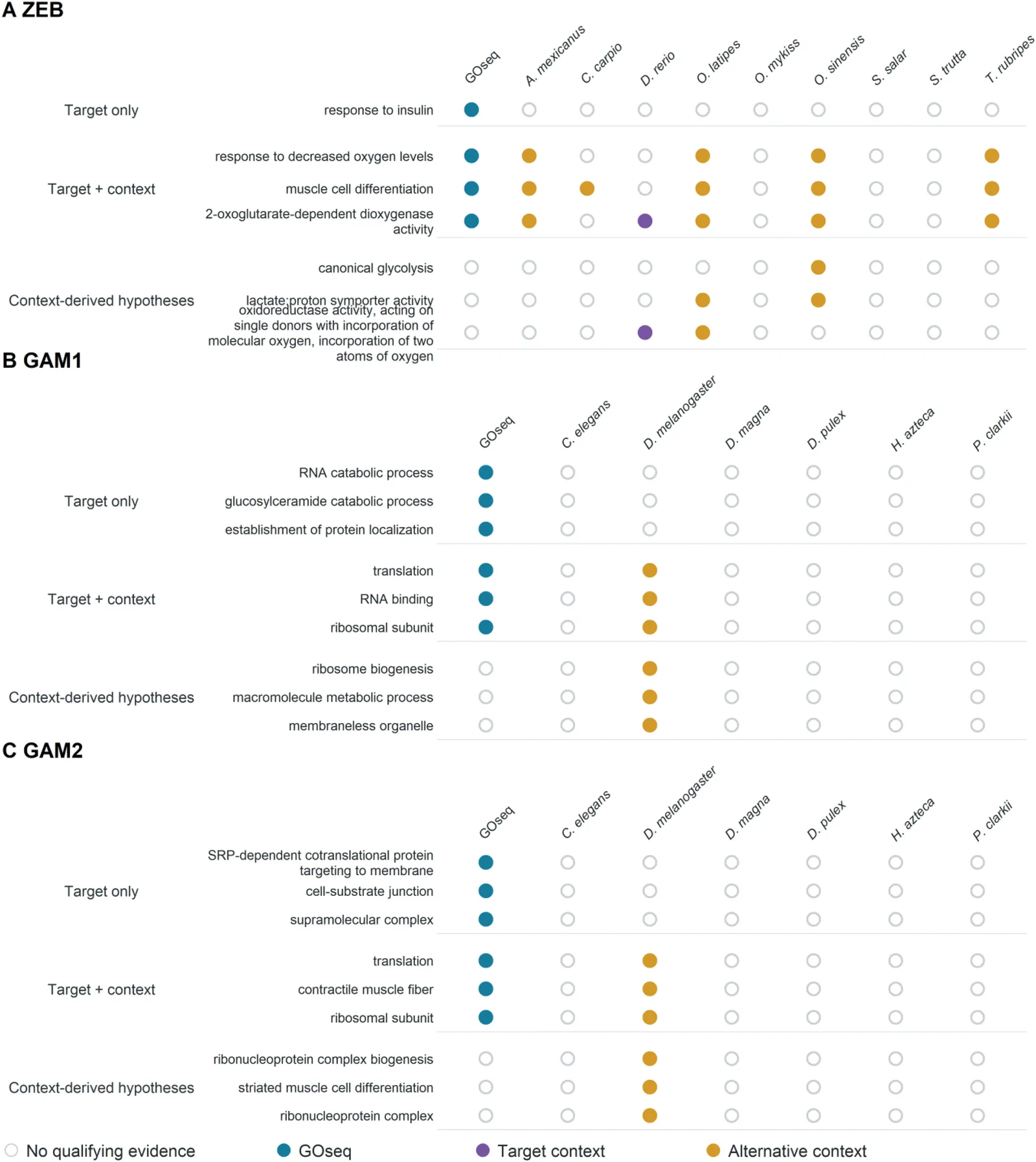
**Annotation-context dependence across the three EchoGO validation exercises.** Representative Gene Ontology (GO) terms are shown for the reference-rich zebrafish exercise (ZEB) and the two annotation-limited *Gammarus pulex* exercises (GAM1 and GAM2). Rows are grouped into three evidence categories: Target only (GO terms supported by the study-specific GOseq analysis but not recovered through alternative contexts), Target + context (GOseq-supported terms also recovered through at least one alternative context), and Context-derived hypotheses (terms recovered through alternative contexts without qualifying GOseq evidence). Columns represent individual enrichment analyses, distinguishing the study-specific GOseq results from g:Profiler analyses using organism-specific annotation resources. Filled circles indicate that the exact GO term meets the significance criterion of the corresponding analysis, whereas open circles indicate no qualifying evidence in that analysis, not biological absence. In ZEB, the zebrafish g:Profiler resource represents the target annotation context, followed by eight alternative fish contexts. GAM1 and GAM2 include six alternative invertebrate contexts but no target-organism g:Profiler context; both retain their study-specific GOseq target analysis. Displayed GO terms represent larger groups of semantically related functions within each evidence category. Representatives were selected from the largest eligible semantic clusters based on their centrality within each cluster, rather than the number of annotation contexts in which they were recovered. The matrices illustrate broad contextual representation of oxygen-response, muscle-development and dioxygenase-related target evidence in ZEB, compared with contextual evidence restricted to the *D. melanogaster* annotation context in GAM1 and GAM2. In the *G. pulex* exercises, this contextual evidence is concentrated on translation- and ribosome-related functions in GAM1 and extends to contractile and muscle-related functions in GAM2. Complete GO-term counts and gene-set representability are reported in Table 2.

The 180 Context-derived hypotheses broadened the functional space accessible through the selected fish resources. Representative examples in Fig. 2A include oxidoreductase activity incorporating two oxygen atoms, canonical glycolysis, and lactate:proton symporter activity, extending the functional picture into related metabolic and transport processes. Their distribution was context-dependent: although the same 92 resolved foreground identifiers were submitted to every contextual analysis, the effective query size varied from 6 to 50 recognised genes among organism resources. Thus, even in this reference-rich exercise, the functional evidence accessible through contextual analysis reflected differences in identifier recognition and annotation coverage among the selected resources.

### Annotation-limited *Gammarus pulex* exercises

The two *G. pulex* exercises provided a contrasting annotation-limited setting. Both originated from the same de novo transcriptome and therefore shared a tested universe of 150,407 Trinity gene-level (_g) features. After identifier resolution and deduplication, this background yielded 5171 unique identifiers for contextual analysis, equivalent to 3.44% of the original tested-feature count. The corresponding foregrounds yielded 239 unique identifiers from 2035 differentially expressed features in GAM1 (11.74%) and 188 from 2093 in GAM2 (8.98%; Table 2). This substantially lower representation of the experimental feature space contrasts with the reference-rich zebrafish exercise.

Within this shared upstream representation constraint, the two comparisons produced different patterns of functional evidence. Target GOseq identified 194 significant GO terms in GAM1, of which 178 were classified as Target only and 16 were also recovered through an alternative annotation context. The Target + context evidence was concentrated around translation- and ribosome-associated functions. This pattern is represented in Fig. 2B by terms including ribosomal subunit, translation, and RNA binding. The 19 Context-derived hypotheses extended this pattern to functions including ribosome biogenesis, macromolecule metabolic process, and membraneless organelle.

GAM2 had fewer significant GOseq terms than GAM1, but a greater proportion were also recovered through alternative annotation contexts. Of its 59 target GOseq terms, 34 were classified as Target only and 25 as Target + context (42.37%), compared with 16 of 194 terms (8.25%) in GAM1. Fig. 2C shows that this Target + context evidence included translation- and ribosome-associated functions but also extended to contractile biology, represented by contractile muscle fibre. Context-derived hypotheses included striated muscle cell differentiation and ribonucleoprotein-complex functions, providing a broader view of the muscle- and protein-synthesis-related response.

Qualifying contextual enrichment evidence in both *G. pulex* exercises was restricted to the *D. melanogaster* annotation context among the six selected invertebrate resources, as shown in Fig. 2B, C. Consequently, the 19 GAM1 and 20 GAM2 Context-derived hypotheses each had a contextual recurrence of one. Together with the low foreground and background resolution, this concentration of contextual evidence illustrates how the accessible functional space in annotation-limited datasets can be shaped by both upstream identifier resolution and the annotation resources available for the selected organism contexts.

### Cross-exercise synthesis

Across the three validation exercises, annotation context influenced functional interpretation in different ways depending on the representation of the experimental feature space and the annotation resources through which it was examined (Table 2; Fig. 2). In the reference-rich ZEB exercise, 42 of 44 target GOseq terms were also recovered through at least one selected annotation context, and several prominent functions were represented across multiple fish resources. Context-derived hypotheses further extended the functional landscape into related metabolic, transport, developmental, and oxygen-associated processes.

The two annotation-limited *G. pulex* exercises showed a different pattern. Only a small fraction of the tested and differentially expressed features could be resolved for contextual analysis, and qualifying contextual evidence was concentrated in the *D. melanogaster* annotation resource. Within this shared constraint, GAM1 and GAM2 nevertheless differed in the functional evidence represented contextually: GAM1 showed contextual recovery primarily around translation and ribosome-associated functions, whereas GAM2 additionally connected target evidence with contractile and muscle-related biology (Fig. 2B, C).

Together, these exercises show that the contribution of alternative annotation contexts depends on both the experimental dataset and its representation within the selected organism resources. In ZEB, contextual analysis revealed that much of the target functional response was represented across several fish annotation resources while also exposing additional hypotheses. In the *G. pulex* exercises, limited identifier representation restricted the accessible contextual feature space, with specific target functions and additional hypotheses represented through the available *D. melanogaster* annotation context. EchoGO makes these differences explicit by retaining the target analysis, contextual evidence, identifier representation, and organism provenance within the same interpretive framework.

### Limitations

EchoGO operates downstream of differential-expression analysis and therefore depends on the experimental feature set, upstream annotation, and available identifier mappings. These factors determine how much of the transcriptomic response can be represented in the foreground and background used for contextual enrichment. This constraint was particularly evident in the *G. pulex* validation exercises, where the 150,407 tested features yielded only 5171 unique identifiers for contextual analysis (3.44% relative to the tested-feature total), while the corresponding foreground identifier yields were 11.74% and 8.98% for GAM1 and GAM2, respectively (Table 2). Improved upstream annotation and identifier resolution can therefore expand the functional feature space accessible to EchoGO.

Functional annotation is also uneven across genes, biological processes, and taxa. Well-characterised and broadly conserved functions may have more opportunities to be represented across multiple organism resources, whereas lineage-specific, rapidly evolving, or sparsely annotated functions may have fewer. Consequently, recurrence across annotation contexts describes the breadth of representation of an exact GO term within the selected resources and is most informative when considered alongside their annotation coverage and biological relevance.

Finally, contextual interpretation depends on the organism annotation resources selected for analysis and on their recognition of the submitted identifiers. In ZEB, effective foreground query sizes ranged from 6 to 50 genes despite submission of the same 92 resolved foreground identifiers, while qualifying contextual evidence in both *G. pulex* exercises was restricted to the *D. melanogaster* resource among the six selected invertebrate contexts. EchoGO records these differences as part of the provenance so that context-derived evidence can be interpreted in relation to the resources through which it was recovered.

## Ethics statements

This article describes a computational workflow and does not involve new experiments on human subjects, vertebrate animals, or social-media-derived data. Packaged example data are provided only for demonstration and evaluation of the workflow.

## Supplementary material *and/or* additional information

EchoGO v0.1.4, including the R package, documentation, demonstration resources, and installation instructions, is available from the public GitHub repository and archived on Zenodo. The validation and reproducibility compendium accompanying this article is deposited separately on Zenodo.

GitHub repository: https://github.com/miloes114/EchoGo

EchoGO v0.1.4: https://doi.org/10.5281/zenodo.23158481

Validation and reproducibility compendium v2.0.0: https://doi.org/10.5281/zenodo.23164928

The validation compendium contains the evidence, provenance, and reproducibility materials for the three validation exercises described in this article (ZEB, GAM1, and GAM2), including the submitted foreground and background vectors, identifier-resolution and annotation-context recognition information, frozen enrichment results, exact-term evidence and provenance, semantic-reduction products, networks, and case-level reports. It also provides the validation summary underlying Table 2 and the complete reproducibility chain for Fig. 2, including the selected-term table, evidence matrix, semantic-selection provenance, generation scripts, and final figure exports. Source enrichment results from the original validation analyses are preserved as frozen evidence, while the accompanying interpretation and reporting products implement the finalised EchoGO v0.1.4 evidence framework. Cached enrichment outputs are retained as part of the reproducibility record because results from online annotation resources may change over time.

## Declaration of generative AI and AI-assisted technologies in the manuscript preparation process

During the preparation of this work, the authors used ChatGPT (OpenAI) to support manuscript organisation, language editing, and revision of the text. After using this tool, the authors reviewed and edited the content as needed and take full responsibility for the content of the publication. OpenAI Codex was used during workflow development to assist with code implementation, debugging, and documentation; all analytical logic, outputs, and final code were reviewed and validated by the authors.

## CRediT authorship contribution statement

**Camilo Escobar-Sierra:** Conceptualization, Methodology, Investigation, Software, Visualization, Formal analysis, Data curation, Validation, Writing – original draft, Writing – review & editing. **Felix Langschied:** Writing – review & editing, Methodology, Validation. **Angelina Miller:** Investigation, Data curation, Writing – review & editing. **Pedro A. Inostroza:** Conceptualization, Supervision, Resources, Funding acquisition, Investigation, Project administration, Writing – review & editing.

## Declaration of competing interest

The authors declare that they have no known competing financial interests or personal relationships that could have appeared to influence the work reported in this paper.

## Data Availability

https://github.com/miloes114/EchoGo
